# Empirical evaluation of scoring functions for Bayesian network model selection

**DOI:** 10.1186/1471-2105-13-S15-S14

**Published:** 2012-09-11

**Authors:** Zhifa Liu, Brandon Malone, Changhe Yuan

**Affiliations:** 1 Department of Computer Science and Engineering, Mississippi State University, Mississippi State, MS 39762, USA; 2 Department of Epidemiology and Public Health, School of Medicine, Yale University, New Haven, CT 06511, USA; 3 Department of Computer Science, Helsinki Institute for Information Technology, Fin-00014 University of Helsinki, Finland; 4 Department of Computer Science, Queens College/City University of New York, Flushing, NY 11367, USA

## Abstract

In this work, we empirically evaluate the capability of various scoring functions of Bayesian networks for recovering true underlying structures. Similar investigations have been carried out before, but they typically relied on approximate learning algorithms to learn the network structures. The suboptimal structures found by the approximation methods have unknown quality and may affect the reliability of their conclusions. Our study uses an optimal algorithm to learn Bayesian network structures from datasets generated from a set of gold standard Bayesian networks. Because all optimal algorithms always learn equivalent networks, this ensures that only the choice of scoring function affects the learned networks. Another shortcoming of the previous studies stems from their use of random synthetic networks as test cases. There is no guarantee that these networks reflect real-world data. We use real-world data to generate our gold-standard structures, so our experimental design more closely approximates real-world situations. A major finding of our study suggests that, in contrast to results reported by several prior works, the Minimum Description Length (MDL) (or equivalently, Bayesian information criterion (BIC)) consistently outperforms other scoring functions such as Akaike's information criterion (AIC), Bayesian Dirichlet equivalence score (BDeu), and factorized normalized maximum likelihood (fNML) in recovering the underlying Bayesian network structures. We believe this finding is a result of using both datasets generated from real-world applications rather than from random processes used in previous studies and learning algorithms to select high-scoring structures rather than selecting random models. Other findings of our study support existing work, e.g., large sample sizes result in learning structures closer to the true underlying structure; the BDeu score is sensitive to the parameter settings; and the fNML performs pretty well on small datasets. We also tested a greedy hill climbing algorithm and observed similar results as the optimal algorithm.

## Introduction

Bayesian networks are compact graphical models for representing uncertain relationships among the random variables in a domain. Often, the relationships are unknown and must be learned from data. A popular approach called score-based learning [1] is to assign a score to each Bayesian network structure according to a scoring function and find the structure that optimizes the score. There are many scoring functions for Bayesian networks, such as minimum description length (MDL) [2] (or equivalently, Bayesian information criterion (BIC) [3]), Akaike's information criterion (AIC) [4], Bayesian Dirichlet equivalence score (BDeu) [5,6], factorized normalized maximum likelihood (fNML) [7], and others [8,9].

The score-based approach to learning Bayesian networks has been shown to be NP-hard [10]; both the running time and memory usage of exact learning are exponential in the number of variables in the worst case. Therefore, early research mainly focused on developing approximation methods [1,11-14]. Recently, however, optimal learning algorithms such as dynamic programming [15-17], branch and bound [18], admissible heuristic search [19-21], and mathematical programming [22,23] have been developed to learn optimal Bayesian networks with several dozens of variables.

Because of the different theoretical underpinnings of these scoring functions, they typically result in different "optimal" networks. Once a scoring function has been selected, though, all optimal algorithms learn equivalent networks; they only differ in running time and memory usage. A major mystery surrounding Bayesian network learning is which scoring function to use given that there are so many choices. Several empirical investigations have been carried out on the performance of various scoring functions in learning Bayesian networks, e.g. [24-26]. These studies, however, have drawbacks in their evaluations because they used local search methods such as K-2 [1] and Greedy Thick Thinning algorithm [27] to select network structures, or even used randomly generated network structures [26]. These suboptimal structures may affect the reliability of their conclusions regarding the model selection capability of the scoring functions. Furthermore, these studies often generate random synthetic networks as the test cases; experimental data thus generated may not share similar properties as real-world data.

In this study, we use an optimal dynamic programming algorithm [16] to learn Bayesian network structures; any other optimal algorithm would yield the same results, however, because only the choice of scoring function affects the learned networks. We study the capability of four scoring functions, MDL, AIC, BDeu, and fNML, to recover the underlying Bayesian network structures. We generated artificial datasets from a set of gold standard Bayesian networks created based on real-world data, learned optimal Bayesian networks for them using different scoring functions, and compared the learned models with the gold standard models based on various evaluation measures. For comparison, we also included the results of a greedy hill climbing algorithm.

Our results offer new insights into the scoring functions in addition to confirming some other common beliefs. In contrast to the results of existing work, a major finding of our study suggests that the MDL/BIC score consistently outperforms AIC, BDeu, and fNML in recovering the underlying Bayesian network structures across various sample sizes. Other findings of our study support existing work. Our results confirm that the structural Hamming distance gives a more reliable measure of the distance between Bayesian net-work structures. We also observed that a parameter selection greatly affects the BDeu score. Finally, it is confirmed that fNML has good performance when the sample sizes are relatively small. Our results using the greedy hill climbing algorithm are similar to those of the optimal learning algorithm, although with higher variances, so our conclusions also hold for the greedy algorithm.

The remainder of this paper is structured as follows. We first review several prior empirical studies of scoring functions. We then provide an overview of Bayesian network and structure learning. After that, we introduce four scoring functions which we will compare. We follow that with a description of the experimental design of this study. Finally, we present the empirical results and discuss our findings.

## Prior work

Several researchers have empirically evaluated the various scoring functions for learning Bayesian networks. In [26], Van Allen and Greiner compared the performance of three different model selection criteria, AIC, BIC, and cross-validation, in finding the right balance between the complexity of the model and the goodness of fit to the training data. First, they randomly generated the gold standard Bayesian network structures as well as the probability parameters. Second, they generated datasets with different sample sizes from the networks. For each dataset, they *again *randomly constructed a set of hypothesis structures and evaluated their quality based on the scoring functions. They found that AIC and cross-validation perform better in avoiding over-fitting in the model selection. While BIC may still work for large sample sizes, it can perform arbitrarily worse than other functions for small datasets. However, they did not use a learning algorithm to try to find good hypothesis structures; they also randomly generated their gold standard networks. It is unclear whether their results stem from the scoring functions or their random model selection technique, or whether the results can be generalized to real-world datasets.

In Yang and Chang's study [24], they compared the performance of five different scoring functions: uniform prior score metric (UPSM), conditional uniform prior score metrics (CUPSM), Dirichlet prior score metric (DPSM), BDe, and BIC. They restricted their experimental evaluations on random networks with three or five nodes as well as a benchmark network called Alarm. Then they generated random datasets from the networks. They used a K2-like search method [1] to learn Bayesian networks. Their greedy structure learning algorithm assumes an ordering over the variables. Then, it greedily adds parents consistent with that ordering to maximize the likelihood of the structure and data set. Because of the ordering assumption and the greedy approach to adding parents, it does not guarantee finding the globally optimal structure. For evaluation, they use the cross-entropy (KL-Divergence) to measure the difference between the learned networks and the true networks. Their results indicated that UPSM, CUPSM, DPSM and BIC are able to correctly identify the true networks. Meanwhile, BDe and DPSM's performance are very sensitive to the *α *value. They may fail to find the true network if the *α *value is not set properly. This study shares the shortcoming of Van Allen and Greiner's study: their gold standard networks are randomly generated, so they may not accurately reflect real-world datasets. Furthermore, their K2-like search method requires an ordering of the variables; in real-world applications, an ordering is often not known *a priori*. Therefore, it is again unclear how their results generalize to real-world situations.

Another related empirical work by de Jongh and Druzdzel [25] investigates structural evaluation measures for Bayesian networks rather than scoring functions. They generated random datasets with different sizes from four benchmark Bayesian networks. Then for each combination of the network and sample size, they ran a local search algorithm called Greedy Thick Thinning [27] to learn Bayesian network structures and calculated the distances between the learned networks and the gold standard networks based on structural Hamming distance, Hamming distance, and other measures. They concluded that the structural Hamming distance is especially useful when looking for the causal structures.

All of these studies have drawbacks in their empirical evaluations. In particular, the conclusions of Van Allen and Greiner are drawn based on randomly generated network structures. Therefore, it is unclear how reliable their conclusions are regarding the model selection capability of the scoring functions. Additionally, the two studies which evaluate scoring functions rely on randomly generated gold standard networks; these may not accurately reflect real-world datasets. The work of de Jongh and Druzdzel only investigates structural evaluation measures using a single scoring function; other scoring functions may behave differently. The current study is designed to address these concerns.

## Bayesian networks

A Bayesian network encodes a joint probability distribution over a set of random variables V = {*X*_1_, ..., *X*_n_}. We consider only discrete variables in this work. Formally, a Bayesian network *B *is a pair {*G*, Θ}, where *G *is a directed acyclic graph (DAG) in which each node corresponds to one of the random variables. The edges or lack of them encode the conditional independence relationships among the variables. The parents of *X*_i _are denoted *P A_i_*; *X_i _*is independent of its non-descendant variables given its parents. Θ specifies the conditional probability distributions *P *(*X_i_*|*P A_i_*) for each *X_i_*. Thus, the joint probability distribution of all of the variables is given as

$$P\left(V\right)=\overset{n}{\underset{i=1}{\prod }}P\left(x_{i}|PA_{i}\right)$$

Given a dataset **D **= {*D*_1_, ..., *D_N _*}, where *D_i _*is an instantiation of all the variables in **V**, Bayesian network structure learning is the problem of learning a network structure from **D**. Assuming *D *is complete and discrete, Θ is maximized using frequency counts from the data [7]. Consequently, finding the optimal Bayesian network reduces to finding the optimal structure.

Score-based learning is a commonly used technique to identify the optimal structure. In this approach, a scoring function is used to measure the goodness of fit of a structure to the data. The goal of the learning problem is then to find the optimally scoring structure. The score typically approximates the probability of the structure given the data and represents a tradeoff between how well the network fits the data and how complex the network is. In this work, we assume the scoring function is *decomposable *[6]. That is, the score for a network structure *B *can be calculated as the sum of scores for the individual variables, where the score for a variable is calculated based solely on the variable and its parents. Therefore,

$$Score\left(B|D\right)=\overset{n}{\underset{i=1}{\sum }}Score\left(X_{i}|PA_{i},D\right),$$

and the learning problem is to find B*, where

$$B^{*}=\underset{B}{\text{arg}\text{max}}\;Score\left(B|D\right).$$

A Bayesian network structure can represent a set of joint probability distributions. Two network structures are said to belong to the same equivalence class if they represent the same set of probability distributions [28]. A scoring function which assigns the same score to networks in the same equivalence class is *score equivalent *[6].

Unfortunately, the number of possible structures is super-exponential in the number of variables; learning an optimal Bayesian network from *D *is shown to be NP-hard [10]. Solving the learning problem exactly becomes impractical if the number of variables is too large. Consequently, much early work focused on approximate algorithms, such as greedy hill climbing approaches [1,11], tabu search with random restarts [13], limiting the number of parents or parameters for each variable [14], searching in the space of equivalence classes of network structures [29], and the optimal reinsertion algorithm (OR) [12]. These algorithms use local search to find "good" networks; however, they offer no guarantee to find the one that optimizes the scoring function. Recently, exact algorithms for learning optimal Bayesian networks have been developed based on dynamic programming [15-17,30,31], branch and bound [18], linear and integer programming (LP) [22,23], and heuristic search [19-21]. These algorithms have enabled us to learn optimal Bayesian networks for datasets with dozens of variables.

Given a scoring function, all optimal learning algorithms learn equivalent networks; hence, the choice of which optimal algorithm is used does not affect the learned network. Consequently, these algorithms make it possible for us to study the behavior of different scoring functions in structure learning without needing to consider the confounding factors resulting from the choice of structure learning algorithms.

## Scoring functions

Many scoring functions are in the form of a penalized log-likelihood (LL) functions. The LL is the log probability of *D *given *B*. Under the standard i.i.d assumption, the likelihood of the data given a structure can be calculated as

$$\begin{array}{llll} LL\left(D|B\right) & =\overset{N}{\underset{j}{\sum }}\text{log}P\left(D_{j}|B\right)\; & & \;\\ & =\overset{n}{\underset{i}{\sum }}\overset{N}{\underset{j}{\sum }}\text{log}P\left(D_{ij}|PA_{ij}\right),\; & & \;\\ & \; & \end{array}$$

where *D_ij _*is the instantiation of *X_i _*in data point *D_j_*, and *PA_ij _*is the instantiation of *X_i_*'s parents in *D_j_*. Adding an arc to a network never decreases the likelihood of the network. Intuitively, the extra arc is simply ignored if it does not add any more information. The extra arcs pose at least two problems, though. First, they may lead to overfitting of the training data and result in poor performance on testing data. Second, densely connected networks increase the running time when using the networks for downstream analysis, such as inference and prediction.

A penalized LL function aims to address the overfitting problem by adding a penalty term which penalizes complex networks. Therefore, even though the complex networks may have a very good LL score, a high penalty term may reduce the score to be below that of a less complex network. Here, we focus on decomposable penalized LL (DPLL) scores, which are always of the form

$$DPLL\left(B,D\right)=LL\left(D|B\right)-\overset{n}{\underset{i=1}{\sum }}Penalty\left(X_{i},B,D\right).$$

There are several well-known DPLL scoring functions for learning Bayesian networks. In this study, we consider MDL, AIC, BDeu and fNML. These scoring functions only differ in the penalty terms, so we will focus on discussing the penalty terms in the following discussions. In terms of memory and runtime, all of the scoring functions incur similar overhead [32].

### Minimum description length (MDL)

The MDL [3] scoring metric for Bayesian networks was defined in [2,33]. MDL approaches scoring Bayesian networks as an information theoretic task. The basic idea is to minimally encode *D *in two parts: the network structure and the unexplained data. The model can be encoded by storing the conditional probability tables of all variables. This requires$\frac{\text{log}\;N}{2}*p$ bits, where $\frac{\text{log}\;N}{2}$ is the expected space required to store one probability value and *p *is the number of individual probability values for all variables. The unexplained part of the data can be explained with *LL*(*D*|*B*) bits. Therefore, we can write the MDL penalty term as

$$PenaltyMDL\left(X_{i},B,D\right)=\frac{\text{log}N*p_{i}}{2},$$

where *p_i _*is the number of parameters for *X_i_*. For MDL, the penalty term reflects that more complex models will require longer encodings. The penalty term for MDL is larger than that of most other scoring functions, so optimal MDL networks tend to be sparser than optimal networks of other scoring functions. As hinted at by its name, an optimal MDL network minimizes rather than maximizes the scoring function. To interpret the penalty as a subtraction, the scores must be multiplied by -1. The Bayesian information criterion (BIC) [3] is a scoring function whose calculation is equivalent to MDL for Bayesian networks, but it is derived based on the asymptotic behavior of the models, that is, BIC is based on having a sufficiently large amount of data. Also, BIC does not require the -1 multiplication.

### Akaike's information criterion (AIC)

Bozdogan [34] defined the AIC [4] scoring metric for Bayesian networks. It, like BIC, is another scoring function based on the asymptotic behavior of models with sufficiently large datasets. In terms of the equation, the penalty for AIC differs from that of MDL by the log *N *term. So the AIC penalty term is

$$PenaltyAIC\left(X_{i},B,D\right)=p_{i}.$$

Because its penalty term is less than that of MDL, AIC tends to favor more complex networks than MDL.

### Bayesian Dirichlet with score equivalence and uniform priors (BDeu)

The Bayesian Dirichlet (BD) scoring function was first proposed by Cooper and Herskovits [1]. It computes the joint probability of a network for a given dataset. However, the BD metric requires a user to specify a parameter for all possible variable-parents combinations. Furthermore, it does not assign the same score to equivalent structures, so it is not score equivalent. To address the problems, a single "hyperparameter" called the *equivalent sample size *was introduced, referred to as *α *[6]. All of the needed parameters can be calculated from *α *and a prior distribution over network structures. This score, called BDe, is score equivalent. Furthermore, if one assumes all network structures are equally likely, that is, the prior distribution over network structures is uniform, *α *is the only input necessary for this scoring function. BDe with this additional uniformity assumption is called BDeu [6]. Somewhat independently, the BDeu scoring function was also proposed earlier by Buntine [5]. BDeu is also a decomposable penalized LL scoring function whose penalty term is

$$PenaltyBDeu\left(X_{i},B,D\right)=\overset{q_{i}}{\underset{j}{\sum }}\overset{r_{i}}{\underset{k}{\sum }}\text{log}\frac{P\left(D_{ijk}|D_{ij}\right)}{P\left(D_{ijk}|D_{ij},\alpha_{ij}\right)},$$

where *q_i _*is the number of possible values of *PA_i_*, *r_i _*is the number of possible values for *X_i_*, *D_ijk _*is the number of times *X_i _*= *k *and *PA_i _*= *j *in *D*, and *α_ij _*is a parameter calculated based on the user-specified *α*. The original derivations [5,6] include a more detailed description. The density of the optimal network structure learned with BDeu is correlated with *α*; low *α *values typically result in sparser networks than higher *α *values. Recent studies [35] have shown the behavior of BDeu is very sensitive to *α*. If the density of the network to be learned is unknown, selecting an appropriate *α *is difficult.

### Factorized normalized maximum likelihood (fNML)

Silander *et al. *developed the fNML score function to address the problem of *α *selection in BDeu based on the normalized maximum likelihood function (NML) [7]. NML is a penalized LL scoring function in which *regret *is the penalty term. Regret is calculated as

$$\underset{D^{\prime }}{\sum }P\left(D^{\prime }|B\right),$$

where the sum ranges over all possible datasets of size *N *. Kontkanen and Myllymäki [36] showed how to efficiently calculate regret for a single variable. By calculating regret for each variable in the dataset, the NML becomes decomposable, or factorized. fNML is given by

$$Penalty\;fNML\left(X_{i},B,D\right)=\overset{q_{i}}{\underset{k}{\sum }}\text{log}C_{N_{ij}}^{r_{i}},$$

where $C_{N_{ij}}^{r_{i}}$ are the regrets. fNML is not score equivalent.

## Methods

Our empirical evaluation of the scoring functions consisted of four phases. First, we created a set of Bayesian networks from real datasets as the gold standard networks. Next, we generated a variety of datasets from each of those gold standard networks by logic sampling. After that, we learned optimal Bayesian networks from the sampled datasets using both an optimal algorithm and a greedy hill climbing algorithm. Finally, we calculated a number of evaluation metrics by comparing the learned networks with the gold standard networks.

### Creating gold standard networks

We need a set of gold standard Bayesian networks as the basis for our empirical evaluations. It is possible to use randomly generated Bayesian networks like several existing studies did, but we want to use models that resemble Bayesian networks that are created for real-world applications. There are many benchmark Bayesian networks available, such as Alarm, CPCS, Hepar, etc., but these benchmark models contain too many variables and are intractable for the current optimal learning algorithms. Therefore, we chose to create the gold standard networks by learning optimal Bayesian networks for a set of UCI machine learning datasets [37] with fewer than 25 variables. This section describes our data processing method for the reproducibility of the results.

The raw UCI datasets contain both continuous and discrete data, as well as missing values. Table 1 describes the detailed information for all the datasets used in this study. Continuous values were discretized using the minimum description length (MDL) discretization technique [38]. MDL discretization recursively partitions a dataset *S *with a single variable *A *by segmenting it into two distinct sets based on a boundary value *T*. The entropy between the two sets is minimal. The entropy between the two sets is defined as

**Table 1:** Summary of gold standard networks

Dataset	Domain	Instances	Nodes	Edges	Average In-degree
Statlog (Australian Credit Approval)	Industry	690	15	33	2.20
Breast Cancer	Biology	699	10	20	2.00
Car Evaluation	Industry	1,728	7	9	1.29
Cleveland Heart Disease	Biology	303	14	22	1.57
Credit Approval	Industry	690	16	35	2.19
Diabetes	Biology	768	9	13	1.44
Glass Identification	Industry	214	10	17	1.70
Statlog (Heart)	Biology	270	14	21	1.50
Hepatitis	Biology	155	20	36	1.80
Iris	Biology	150	5	8	1.60
Nursery	Industry	12,960	9	14	1.56
Statlog (Vehicle Silhouettes)	Industry	846	19	40	2.11
Congressional Voting Records	Political	436	17	46	2.71

This table describes all of the datasets we used in this study. *Dataset *gives the name of the dataset in the UCI machine learning repository. *Domain *gives a rough indication of the domain of the dataset. *Instances *gives the number of instances in the original dataset. *Nodes *gives the number of variables in the dataset (and the number of nodes in the corresponding Bayesian network). *Edges *gives the number of edges in the optimal Bayesian network learned from the original dataset. This is the gold standard network used throughout the rest of the evaluation. *Average In *- *degree *gives the average number of parents of each variable in the learned Bayesian network.

$$E=\frac{\left|S_{1}\right|}{\left|S_{2}\right|}Ent\left(S_{1}\right)+\frac{\left|S_{2}\right|}{\left|S_{1}\right|}Ent\left(S_{2}\right),$$

where *S*_1 _and *S*_2 _are the segments of *S *based on partitioning at *T *and *Ent*(·) is the entropy of the single set.

The recursion stops when the information gain of adding another partition does not exceed the cost of encoding the two new separate classes, given as

$$\begin{array}{llll} Gain & >\frac{log_{2}\left(|S|-1\right)}{\left|S\right|}+\frac{\Delta \left(A,T;S\right)}{\left|S\right|},\; & & \;\\ \Delta \left(A,T;S\right) & =log_{2}\left(3^{k}-2\right)+k\times Ent\left(S\right)\; & & \;\\ & \;-k_{1}\times Ent\left(S_{1}\right)-k_{2}\times Ent\left(S_{2}\right)\; & & \;\\ & \; & \end{array}$$

where *k_i _*is the number of distinct values of *A *in *S_i_*.

Although the MDL discretization technique has the same theoretical basis as the MDL scoring function, it is otherwise unrelated. That is, using the MDL discretization does not favor the MDL scoring function over the others in any way.

We used a *k *nearest neighbors (kNN) algorithm to impute missing values [39]. The kNN algorithm computes a missing value *X_p _*for record *D_i _*by finding the *k *closest *D_j_*s (out of those records which are not missing any values) to *D_i _*(using Euclidean distance, for example), excluding *X_p_*. If *X_p _*is a continuous variable, the value of *X_p _*is averaged for each of the *D_j_*s, and that value is assigned to *X_p _*for *D_i_*. If categorical, it is replaced by a majority vote among the *k *closest neighbors for *X_p_*. We set *k *= 5.

After processing the datasets, we applied an optimal learning algorithm based on the MDL scoring function [17] to learn optimal Bayesian networks. Again, the use of MDL score here does not affect the conclusions of this study, as other scoring functions yielded similar results. We used the maximum likelihood estimation method to learn the parameters of the networks. We took the learned networks as the gold standard networks and generated datasets from them.

### Generating datasets from gold standard networks

After we created the gold standard networks, we generated datasets for each of these Bayesian networks with different numbers of data points ranging from 200 and 1000 (with increments equal to 200) and from 1,000 and 10,000 (with increments equal to 1,000), for a total of 18 sample sizes for each gold standard network. Each data point in a dataset corresponds to one random sample drawn from the joint probability distribution of a Bayesian network using logic sampling [40]. The basic idea is to sample the value for each variable according to the conditional probability distribution of the variable given its parents. The sampling is performed in a topological order of all the variables in order that all the parents already have sampled values before the child variable is sampled.

### Learning from the sampled datasets

After generating datasets from the gold standard networks, we learned optimal networks for all the datasets by using the aforementioned scoring metrics. MDL, AIC and fNML are parameterless, so we learned one network for each combination of scoring function and dataset. All optimal learning algorithms would learn an equivalent network, so our choice of optimal learning algorithm does not affect the learned network. We tried the following *α *values, 0.1, 0.5, 1, 5, 10, 20, 50, 80, 100, for the hyperparameter *α *of BDeu and learned a network for each combination of *α *value and dataset. Thus, in total, we learned 12 "optimal" networks for each dataset and sample size. For comparison, we also tested a greedy hill climbing algorithm with random restarts and a tabu list in the same experiments.

### Evaluating the learned networks

We used several structural evaluation metrics to compare the performance of the different scoring functions. Three of the evaluation metrics operate directly on the gold standard and learned DAG structures: *accuracy, sensitivity*, and *average hamming distance *(AHD). The formulas for those metrics are

$$\begin{array}{llll} Accuracy & =\frac{TP+TN}{TP+TN+FP+FN},\; & & \;\\ Sensitivity & =\frac{TP}{TP+FN},\; & & \;\\ AHD & =\frac{FP+FN}{n},\; & & \;\\ & \; & \end{array}$$

where a *TP *is an edge in the correct direction in the learned network, a *TN *is an edge in neither the learned nor the gold standard network, a *FP *is an edge in the learned network but not in the gold standard network, and a *FN *is an edge in the gold standard but not in the learned network. Note that an edge in the wrong direction in the learned network counts as both a *FP *and a *FN*.

We also used an evaluation metric called *structural Hamming distance *(SHD). As mentioned earlier, multiple structures with edges in different directions may belong to the same equivalence class. Intuitively, the distance between Bayesian networks in the same equivalence class should be zero. To accommodate this, SHD first identifies the equivalence class to which a Bayesian network belongs using an algorithm given by Chickering [28]. An equivalence class is represented by a partially directed graph (PDAG) in which some edges are directed and some undirected. The undirected edges can be orientated arbitrary as long as no new V structure in which multiple variables share a child is introduced. SHD then counts the number of directed and undirected edge additions, deletions, reversals and changes in direction to transform one PDAG into the other as the distance between two corresponding Bayesian networks. Tsamardinos *et al. *[41] provide a more formal algorithm for computing the SHD metric.

## Results

In this section, we present the results of our empirical study. We first compared the evaluation metrics in order to select one metric for further analysis. We next looked into the effect of the hyperparameter *α *on the BDeu score. We then compared the capability of the scoring functions in recovering the Bayesian network structures from the sampled datasets generated from the gold standard Bayesian networks. After that, we compared the effect of sample sizes on the performance of the scoring functions in learning from the datasets when using both an optimal learning algorithm and a greedy hill climbing algorithm.

### Comparison of evaluation metrics

**Figure 1:**
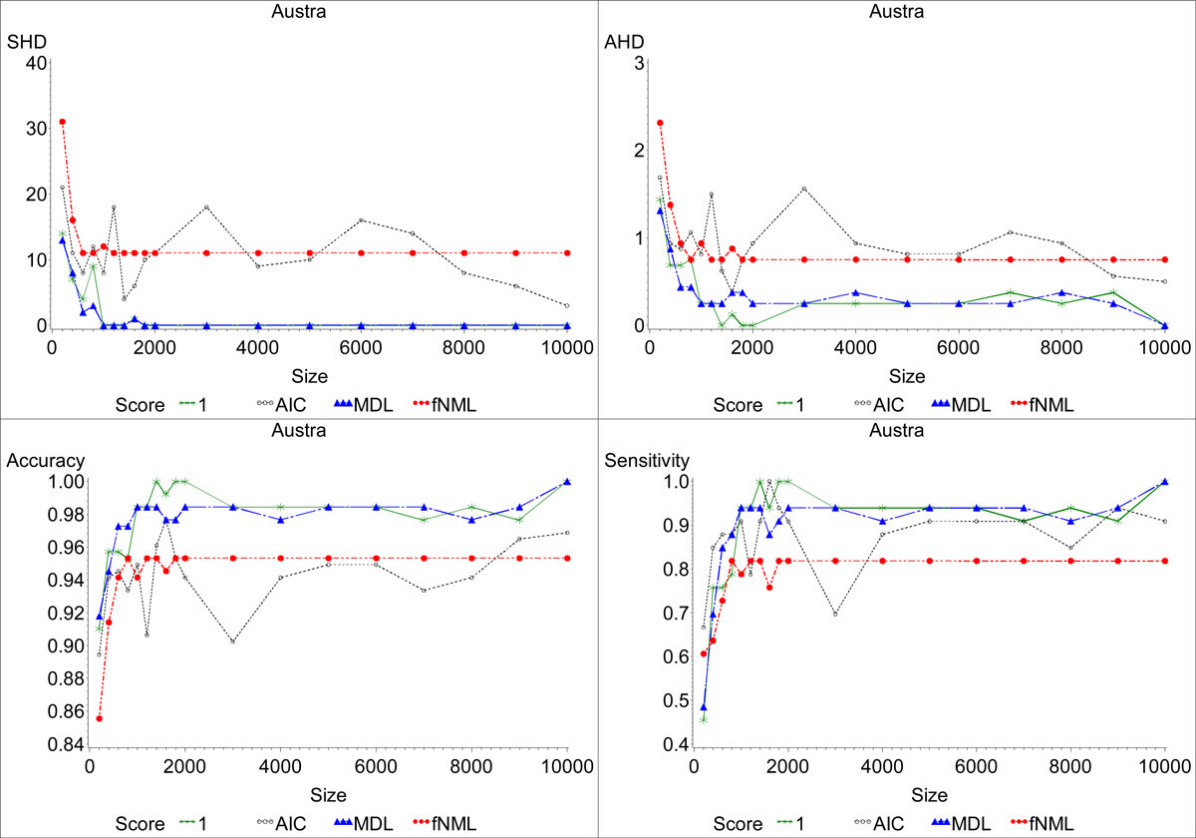
**Comparing the evaluation measures for the optimal networks learned from the Austra datasets with different sizes**. In this figure, we compare the performance of the four evaluation metrics (SHD, AHD, accuracy, and sensitivity) for the *Australian Credit Approval *dataset. The y-axis label indicates which evaluation metric that graph displays. We display the results for *α *= 1 for BDeu for all measures because it had the best convergence behavior for this dataset. We used the behavior of each of the curves to evaluate the convergence of the corresponding scoring function. We consider a scoring function to have converged for an evaluation metric when increasing the dataset size does not change the value for that scoring function and evaluation metric. Thus, we look for "flat lines" in the graphs.

**Figure 2:**
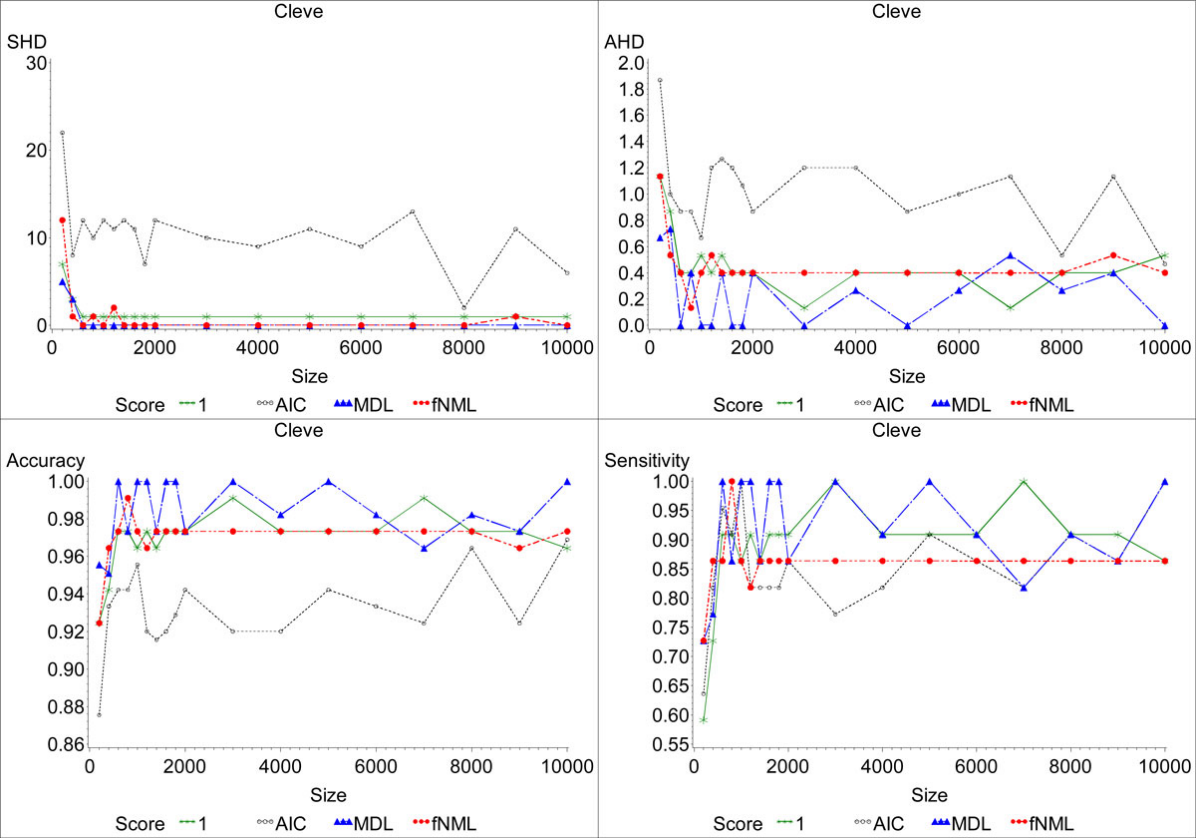
**Comparing the evaluation measures for the optimal networks learned from the Cleve datasets with different sizes**. In this figure, we compare the performance of the four evaluation metrics (SHD, AHD, accuracy and sensitivity) for the *Cleve *dataset.

We first compared the robustness of the evaluation measures as the sample size increases in the datasets. Theoretically, as the number of data points increases, the bias introduced by the penalty term in a scoring function has decreasing effect, and the learned model should gradually converge to the equivalence class of the true underlying model [29]. Figures 1 and 2 show the convergence results for the scoring functions on the optimal networks learned for the *Statlog *(*Australian CreditApproval*) and *Cleveland Heart Disease *datasets respectively. We consider an evaluation measure to have *converged *when adding more data points does not change the value of the metric. Our results show that the SHD metric converges for most of scoring functions with a small number of data points. In contrast, AHD, accuracy and sensitivity still fluctuate when there is a large number of samples. We only show the results on two datasets, but the results on the other datasets are similar. SHD exhibits better convergence behavior because it operates on the equivalence classes of networks rather than directly on the specific DAGs in question. As a simple example, suppose the gold standard network is *X *→ *Y*, but the learned network is *X *← *Y*. The two networks represent the same conditional independencies, and SHD gives a distance of 0. However AHD, accuracy, and sensitivity all consider the arc incorrect because the arcs are oriented in different directions. We therefore only use SHD for the rest of our analysis.

### BDeu parameterizations

**Figure 3:**
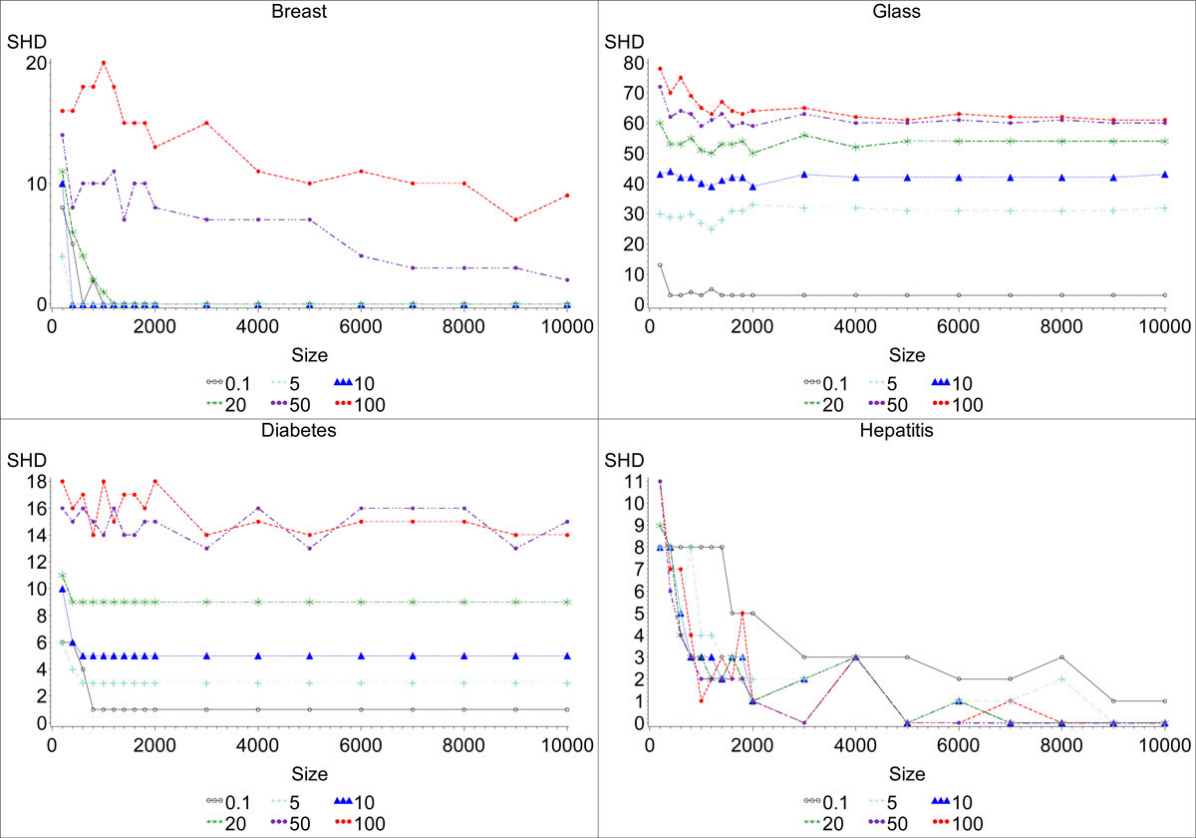
**The effect of the hyperparameter *α *on the BDeu score**. This figure plots the SHD between the networks learned by BDeu and the gold standard networks for six values of *α *for the *Breast*, *Glass*, *Diabetes*, and *Hepatitis *datasets. We used the behavior of each curve to evaluate both the convergence and the recovery ability of each value of *α*. We evaluate the recovery ability by considering both the smallest SHD for the scoring function, the size of the dataset which gives that SHD, and whether the scoring function converged to the smallest SHD, some other SHD or did not converge.

We also investigated the effect of the hyperparameter *α *on BDeu. We focused on both the convergence behavior and the effect of *α *on recovering the gold standard networks. The results are shown in Figure 3 and Table 2. While some *α *values give good recovery results, it is clear that selecting either too low or too high of an *α *can dramatically impact the quality of the learned networks. BDeu was similarly impacted by *α *on other datasets as shown in the Additional File 1 S1.xls (sheet = results . optimal). On some of the networks, a poorly chosen *α *value may prevent convergence of the algorithms even when the sample size is large. As mentioned earlier, low *α*s tend to result in sparser networks than higher *α*s. Unfortunately, if the density of the gold standard network is unknown, selecting *α *is difficult. Consequently, BDeu is only a good scoring function if an expert can appropriately estimate *α*. Otherwise, the learned network is either too sparse (if *α *is too low) or too dense (if *α *is too high). This analysis supports previously published results [35].

**Table 2:** Summary of the effect of different *α *values on the performance of BDeu

	α = 0.1				*α *= 0.5				*α *= 1			
**GoldNet**	**Min**	**Mean**	**Max**	**STD**	**Min**	**Mean**	**Max**	**STD**	**Min**	**Mean**	**Max**	**STD**

Austra	0	2.44	14	4.38	0	2.11	14	4.32	0	1.94*	14	4.02
Breast	0	0.83	8	2.18	0	0.61	5	1.50	0	0.61	5	1.50
Car	0	1.44	5	2.28	0	0.89	5	1.91	0	0.89	5	1.91
Cleve	1	1.83	11	2.43	1	1.50	7	1.54	1	1.44*	7	1.46
Crx	3	5.72*	18	4.56	3	6.06	19	5.46	3	5.72	18	4.91
Diabetes	1	1.72	6	1.71	1	1.22*	4	0.73	1	1.28	4	0.75
Glass	1	1.83	7	2.01	1	1.00*	1	0.00	1	1.00*	1	0.00
Heart	0	0.00*	0	0.00	0	0.00	0	0.00	0	0.00	0	0.00
Hepatitis	3	3.72*	13	2.37	3	4.22	12	2.62	6	7.06	13	2.01
Iris	1	1.83	7	1.72	1	1.44	6	1.34	1	1.33*	4	0.97
Nursery	1	4.94	8	2.75	0	4.39	8	2.77	0	4.06	8	2.92
Vehicle	0	0.67	9	2.14	0	0.22	4	0.94	0	0.22*	4	0.94
Voting	0	1.61	23	5.38	0	1.39	22	5.17	0	1.28*	19	4.47

	**α = 5**				***α *= 10**				***α *= 20**			

**GoldNet**	**Min**	**Mean**	**Max**	**STD**	**Min**	**Mean**	**Max**	**STD**	**Min**	**Mean**	**Max**	**STD**

Austra	0	3.61	18	5.81	1	13.94	21	3.81	14	15.39	25	3.11
Breast	0	0.22*	4	0.94	0	0.56	10	2.36	0	1.33	11	2.93
Car	0	0.28*	5	1.18	0	0.28	5	1.18	0	0.33	5	1.19
Cleve	4	6.61	13	2.85	9	12.56	19	2.18	20	21.56	25	1.10
Crx	5	13.72	21	5.49	13	16.83	20	1.86	18	20.17	29	2.90
Diabetes	3	3.22	6	0.73	5	5.33	10	1.19	9	9.11	11	0.47
Glass	7	7.89	8	0.32	12	14.67	15	0.84	18	19.83	20	0.51
Heart	1	1.00	1	0.00	1	1.44	2	0.51	2	2.22	4	0.55
Hepatitis	25	30.22	33	1.99	39	41.78	44	1.31	50	53.56	60	2.25
Iris	3	3.22	5	0.55	5	5.22	8	0.73	9	9.61	14	1.38
Nursery	0	3.17	8	2.64	0	2.50	8	2.48	0	2.39*	9	2.57
Vehicle	0	0.44	5	1.20	1	2.39	10	2.03	5	6.72	15	2.47
Voting	0	1.61	22	5.14	0	3.89	30	7.06	0	7.06	38	9.05

	**α = 50**				***α *= 80**				***α *= 100**			

**GoldNet**	**Min**	**Mean**	**Max**	**STD**	**Min**	**Mean**	**Max**	**STD**	**Min**	**Mean**	**Max**	**STD**

Austra	16	19.67	33	4.35	18	22.50	42	5.99	19	24.17	42	5.86
Breast	2	7.44	14	3.35	7	11.67	16	3.36	7	13.72	20	3.72
Car	0	0.67	8	1.88	0	1.50	8	1.76	0	1.94	8	1.66
Cleve	26	27.50	34	1.95	27	29.72	41	3.27	28	30.50	42	3.49
Crx	19	25.39	37	4.41	24	29.28	40	4.08	27	31.78	44	4.35
Diabetes	13	14.89	16	1.13	14	16.33	18	1.37	14	15.67	18	1.50
Glass	18	18.11	20	0.47	18	21.61	26	2.06	20	24.44	26	2.04
Heart	4	4.11	5	0.32	4	4.61	5	0.50	4	4.94	5	0.24
Hepatitis	59	61.50	72	3.03	61	64.28	75	3.92	61	65.28	78	4.86
Iris	13	15.06	18	1.35	14	16.78	18	1.11	14	15.83	18	1.47
Nursery	0	2.11	11	2.78	0	2.33	10	2.81	0	2.61	11	3.13
Vehicle	14	18.44	30	3.88	19	23.50	36	4.08	22	27.44	39	4.15
Voting	6	22.28	43	9.78	16	30.00	52	8.60	23	34.50	56	8.21

This table shows SHD statistics about the networks learned using the sampled datasets for the BDeu scoring function for all of the *α *values that we analyzed. *GoldNet *gives the name of the network. We have used abbreviated names from Table 1, but the order of the datasets is the same in both tables. *Min*, *Mean*, *Max *and *STD *give the particular statistic for SHD for all sample sizes for the given network and *α *value. The *α *value with the lowest mean for each dataset is shown in bold and marked with "*".

### Gold standard network recovery

**Figure 4:**
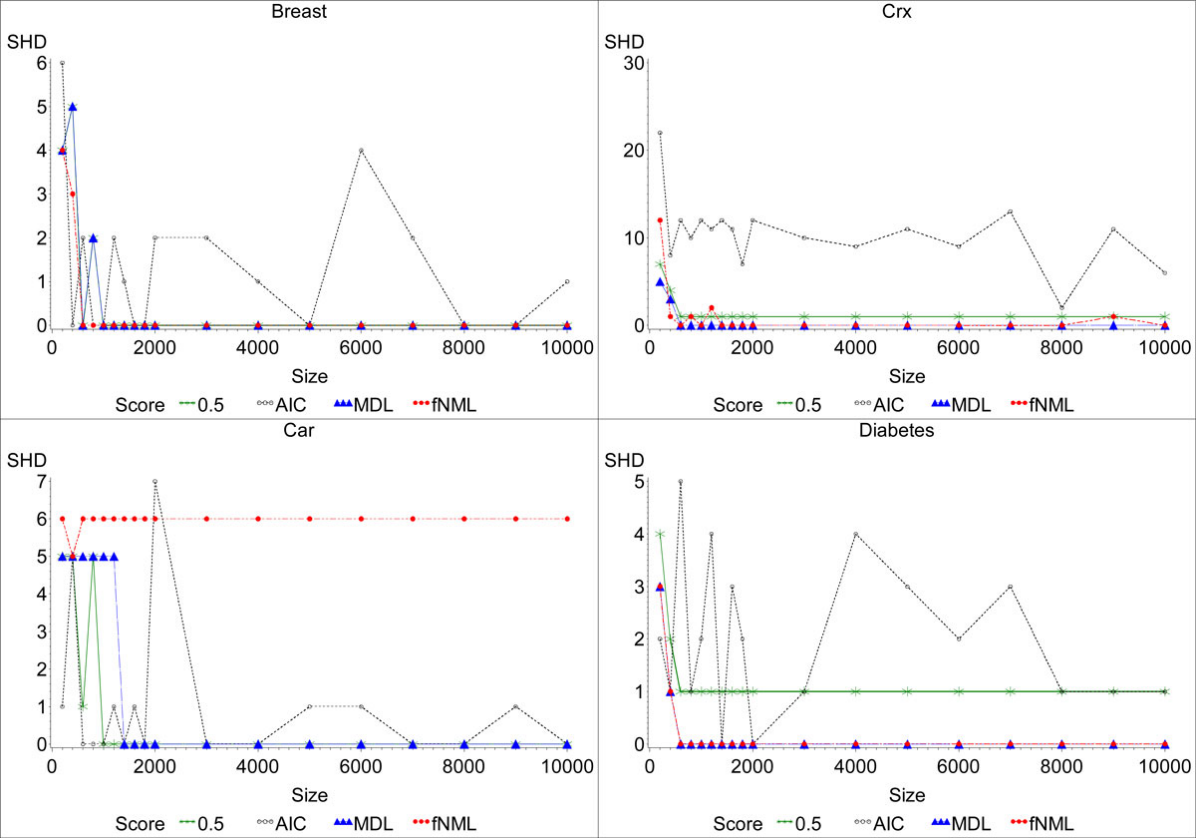
**Plot of structural Hamming distance of the networks learned by optimal learning algorithm from datasets with different sample sizes**. This figure plots the SHD of the networks learned by each of the scoring functions for the *Breast*, *Crx*, *Car*, and *Diabetes *datasets. We display the results for *α *= 0.5 for BDeu for all datasets because it had the best behavior in terms of SHD.

We studied the capability of each scoring function in recovering the gold standard network based on the SHD metric. In the case of BDeu, we show the behavior of the best performing *α *value. Figure 4 shows that most of the scoring functions can recover the gold standard network on four of the datasets given a large enough sample size and appropriate parameters (*α *for BDeu). Other datasets exhibit similar behavior as shown in Table 3 and the Additional file 1 S1.xls (sheet = results . optimal). In particular, we consider the minimum distance of each scoring function and dataset. A minimum distance of 0 means that the gold standard network was recovered for the dataset. Small distances indicate that the scoring function guided the learning algorithm to find close to optimal networks.

**Table 3:** A comparison of the performance of four scoring functions in recovering the true underlying Bayesian network structures

	AIC				MDL				fNML				BDeu			
**GoldNet**	**Min**	**Mean**	**Max**	**STD**	**Min**	**Mean**	**Max**	**STD**	**Min**	**Mean**	**Max**	**STD**	**Min**	**Mean**	**Max**	**STD**

Austra	3	10.72	21	5.02	0	1.50*	13	3.49	11	12.44	31	4.78	0	1.94	14	4.02
Breast	0	1.28	6	1.64	0	0.61	5	1.50	0	0.39	4	1.14	0	0.22*	4	0.94
Car	0	1.00	7	1.91	0	1.67	5	2.43	5	5.94	6	0.24	0	0.28*	5	1.18
Cleve	2	10.44	22	3.94	0	0.44*	5	1.34	0	0.94	12	2.82	1	1.44	7	1.46
Crx	9	15.28	24	4.20	3	4.67*	18	3.79	13	14.44	34	4.94	3	5.72	18	4.56
Diabetes	0	2.00	5	1.41	0	0.22*	3	0.73	0	0.22*	3	0.73	1	1.22	4	0.73
Glass	0	0.00*	0	0.00	0	0.00*	0	0.00	0	0.06	1	0.24	1	1.00	1	0.00
Heart	0	0.00*	0	0.00	0	0.00*	0	0.00	0	0.00*	0	0.00	0	0.00*	0	0.00
Hepatitis	17	21.83	31	4.13	0	0.44*	6	1.46	0	2.94	24	5.95	3	3.72	13	2.37
Iris	0	1.78	5	1.80	0	0.33	3	0.97	0	0.17*	3	0.71	1	1.33	4	0.97
Nursery	0	3.61*	12	3.99	0	4.94	8	3.28	8	9.22	16	2.18	0	4.39	8	2.77
Vehicle	0	0.72	4	1.07	0	1.11	16	3.77	0	0.39	7	1.65	0	0.22*	4	0.94
Voting	8	14.61	32	6.15	0	1.11*	16	3.77	0	2.50	31	7.42	0	1.28	19	4.47

This table shows SHD statistics about the networks learned using the sampled datasets for all scoring functions that we analyzed. For BDeu, we used the value of *α *that gave the lowest mean SHD. *GoldNet *gives the name of the network. We have used abbreviated names from Table 1, but the order of the datasets is the same in both tables. *Min*, *Mean*, *Max *and *STD *give the particular statistic for SHD for all sample sizes for the given network and scoring function. The scoring function with the lowest mean for each dataset is shown in bold.

In contrast to the results reported by several previous studies, we found that MDL was able to recover the gold standard network more quickly than other scoring functions. We observe these differences both because we use an optimal learning algorithm and because we use gold standard networks representing real-world datasets. Given an appropriate *α *value, BDeu also converged to the gold standard networks within the sample sizes we tested. In some of the datasets, fNML converged to the gold standard network very quickly, but sometimes it converged to a different network. In contrast, AIC's behavior was much more erratic. It found the gold standard network on 8 of the datasets. But because of its high standard deviation, we infer it never completely converged. Figure 4 supports this conclusion. In light of these results, we conclude that MDL is a good scoring function because it often converges to the gold standard network. BDeu also exhibits good behavior if a suitable *α *is known before learning.

### Convergence behavior

Next, we studied the convergence behavior of each scoring function. We did not consider whether the scoring function converged to the gold standard network; rather, we only focused on whether the scoring function converged to *any *network. In essence, this part of our study investigates the effect of the size of a dataset on the scoring functions. We again consult Figure 4 and Table 3 but this time look for convergence of the scoring functions; that is, we look to see at what point increasing sampling size does not change SHD anymore. As the figure shows, most of the scoring functions converged. To look for convergence in the table, we consider the mean, minimum, maximum, and standard deviation for the SHD statistics. We expect that if the scoring function converged quickly, its standard deviation will be small. This loose interpretation is robust in that it allows us to conclude that a scoring function converged even if SHD changes slightly from one sample size to the next.

As previously shown [7], fNML converges with fewer samples than the other scoring functions. Because the mean SHD is typically small, we conclude that the network to which it converges is often close to the gold standard network. MDL converged somewhat more slowly, but often converged to the gold standard network. BDeu with an optimal *α *value tends to converge quickly to a network close to the gold standard networks; however, with a sub-optimal *α *value, BDeu often neither converges nor comes close to the gold standard networks as shown in Table 2. Because AIC has a very low penalty term, more data encourages it to add more edges. Thus, it tends to overfit the data on large sample sizes and rarely converges. The SHD of AIC does tend to decrease as the sampling size increases, but that trend is somewhat inconsistent. Based on these results, fNML seems to be a good scoring function when data is limited, while MDL is superior when more data is present.

### Comparison to greedy hill climbing

Finally, we compared the network recovery and convergence ability of a greedy hill climbing learning algorithm to those from the optimal algorithm. We performed this analysis because, as mentioned, optimal learning algorithms are limited to datasets with several dozens of variables. While some biological datasets (such as the *Breast Cancer*, *Cleveland Heart Database*, *Diabetes*, *Statlog *(*Heart*), *Hepatitis *and *Iris *datasets included in this study) are within this limit, many others, such as gene expression datasets, include hundreds or thousands of variables. Greedy hill climbing algorithms have been shown to scale to datasets of this size [14]. This part of our study verifies that our conclusions on scoring functions apply to this algorithm, as well.

We first evaluated the network recovery ability of the scoring functions on the greedy hill climbing algorithm. Table 4 shows that, much like the optimal learning algorithms, the hill climbing algorithm typically either adds extra edges or misses necessary edges. On the other hand, as the small values in the *Reverse *and *Compelled *columns show, the directionality of the edges is typically correct. The *Total *SHD follows a similar trend among the greedy hill climbing and optimal algorithms. That is, scoring functions that performed well for the optimal algorithm also performed well for the hill climbing algorithm. We observed similar results on the other datasets as shown in the Additional file 1 S1.xls (sheet = results . greedy). These results confirm that the scoring functions have a similar impact on structure recovery regardless of whether an optimal or greedy algorithm is used. In almost all cases, though, the optimal algorithm finds a structure closer to the true gold standard networks, so its *Total *distance is always lower. This highlights the benefit of using optimal algorithms when possible.

**Table 4:** A Comparison of Structural Error for the suboptimal learning algorithm and the optimal learning algorithm

			Greedy Hill Climbing					Optimal				
**GoldNet**	**Size**	**Score**	**Add**	**Delete**	**Rev**	**Mis**	**Total**	**Add**	**Delete**	**Rev**	**Mis**	**Total**

Austr	200	AIC	16	14	1	1	32	11	6	2	2	21
	200	MDL	9	17	0	0	26	0	8	1	4	13
	200	fNML	11	16	0	1	28	20	7	0	4	31
	200	0.1	7	17	0	1	25	0	10	0	4	14
	200	0.5	9	17	0	0	26	1	9	1	3	14
	200	1	9	17	0	0	26	1	9	1	3	14
	200	5	11	12	2	2	27	5	6	1	6	18
	200	10	14	14	0	2	30	8	7	2	4	21
	600	AIC	18	15	1	0	34	7	1	0	0	8
	600	MDL	13	15	1	0	29	0	2	0	0	2
	600	fNML	13	15	2	0	30	1	3	0	7	11
	600	0.1	11	15	1	1	28	0	4	0	1	5
	600	0.5	12	15	1	1	29	0	3	0	1	4
	600	1	12	15	1	1	29	0	3	0	1	4
	600	5	14	14	1	4	33	1	2	0	0	3
	600	10	15	15	0	3	33	4	3	1	9	17
	1000	AIC	18	13	1	0	32	7	0	1	0	8
	1000	MDL	15	15	1	0	31	0	0	0	0	0
	1000	fNML	16	15	0	3	34	2	1	1	8	12
	1000	0.1	15	15	1	0	31	0	0	0	0	0
	1000	0.5	15	15	1	0	31	0	0	0	0	0
	1000	1	15	15	1	0	31	0	0	0	0	0
	1000	5	17	15	2	1	35	2	0	4	6	12
	1000	10	18	15	2	1	36	4	1	1	8	14

Crx	200	AIC	20	14	0	2	36	9	2	4	3	18
	200	MDL	9	16	0	3	28	1	8	0	9	18
	200	fNML	16	15	1	1	33	19	5	6	4	34
	200	0.1	6	16	0	3	25	1	11	0	6	18
	200	0.5	10	16	0	3	29	1	8	0	9	18
	200	1	9	15	0	4	28	1	7	0	10	18
	200	5	13	14	1	2	30	5	6	3	5	19
	200	10	19	14	2	0	35	9	4	3	3	19
	600	AIC	21	14	0	0	35	8	1	2	0	11
	600	MDL	14	16	0	0	30	1	3	1	0	5
	600	fNML	14	14	0	4	32	3	3	1	7	14
	600	0.1	11	15	0	1	27	2	6	2	1	11
	600	0.5	13	15	0	0	28	1	3	1	0	5
	600	1	13	15	0	0	28	1	3	1	0	5
	600	5	17	13	2	3	35	6	2	2	7	17
	600	10	18	13	0	3	34	8	3	2	6	19
	1000	AIC	21	15	0	0	36	7	1	1	0	9
	1000	MDL	14	15	1	0	30	1	2	1	1	5
	1000	fNML	17	15	0	4	36	2	2	0	9	13
	1000	0.1	14	15	0	0	29	1	3	1	1	6
	1000	0.5	13	15	0	0	28	1	3	1	1	6
	1000	1	13	15	0	0	28	1	3	1	1	6
	1000	5	17	15	0	0	32	4	2	0	11	17
	1000	10	18	14	2	4	38	6	2	1	8	17

This table gives detailed information about the structural differences between the learned and gold standard networks for the *Statlog *(*Australian Credit Approval*) and *Credit Approval *datasets. It shows differences for both the greedy hill climbing and the optimal learning algorithm. *GoldNet *gives the name of the network. *Size *gives the sample size. *Score *gives the scoring function. When only a number is shown, the scoring function is BDeu with that value for *α*. *Add *gives the number of edges that were added to the learned network that were not in the gold standard network. *Delete *gives the number of edges that were not in the learned network which were in the gold standard network. *Rev *gives the number of edges that were oriented in the wrong direction in the equivalence class of the learned network compared to that of the gold standard network; that is, the number of edges that were *reversed*. *Mis *gives the number of edges that were either directed in the equivalence class of the learned network and undirected in that of the gold standard network, or vice versa; that is, it gives the number of *mis*-directed edges.

**Figure 5:**
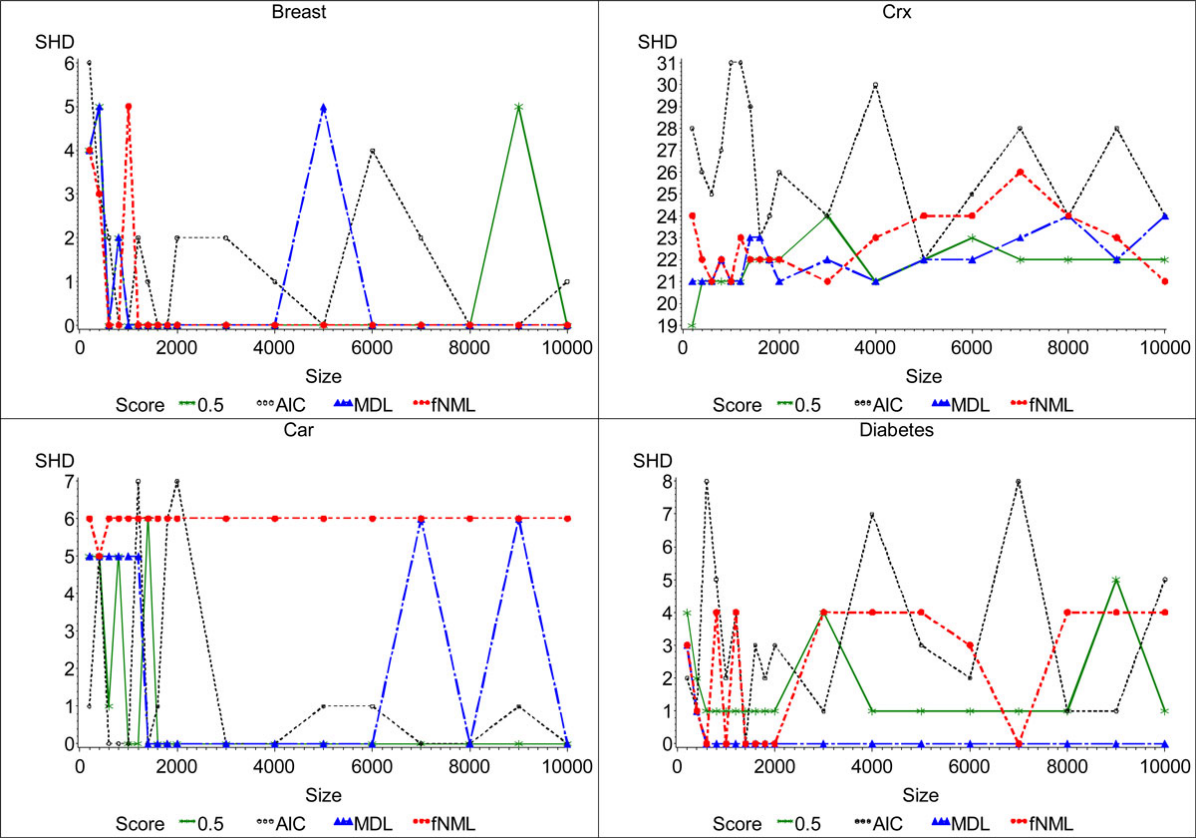
**Plot of structural Hamming distance of the networks learned by the sub-optimal learning algorithm from datasets with different sample sizes**. This figure plots the SHD of the networks learned by each of the scoring functions for the *Breast*, *Crx*, *Car*, and *Diabetes *datasets. We display the results for *α *= 0.5 for BDeu for all datasets because it had the best behavior in terms of SHD.

We then evaluated the convergence behavior of the scoring function on the greedy hill climbing algorithm. As shown in Figure 5, the picture is not as clear as the convergence behavior of the optimal algorithm in Figure 4. Nevertheless, we still see similar trends. Of the scoring functions, fNML typically converges the quickest, though often to a worse network than MDL. On the *Breast Cancer *and *Car Evaluation *datasets, MDL converges to the gold standard network, except for a few perturbations caused by the uncertainty of the greedy search algorithm. BDeu also converges except for a few spikes, but it typically converges to a worse network than MDL. As with the optimal algorithm, AIC does not converge. These results also mirror those of the behavior we observed in the optimal algorithm, though a bit noisier. They again suggest that the conclusions we drew from the optimal algorithms apply to the greedy algorithm, albeit with some noise. We also see that the optimal algorithm gives more consistent behavior, both in terms of quality and consistent convergence, and should be used when possible.

## Conclusion

In this work, we have empirically investigated the ability of four Bayesian network scoring functions (MDL, AIC, BDeu and fNML) to recover the generating distribution of a dataset; a gold standard Bayesian network represents this distribution. We used an optimal structure learning algorithm to ensure approximation algorithms did not affect the learned networks. All optimal learning algorithms would learn an equivalent network, so our choice of optimal algorithm did not affect our results or conclusions. Then, we controlled scoring function and sample sizes to test their effect on the quality of the learned networks. We also considered four different evaluation metrics: accuracy, sensitivity, AHD and SHD. In addition, we evaluated a greedy hill climbing algorithm to ensure that our conclusions are valid for algorithms which can learn networks with hundreds or thousands of variables.

As a result of our investigation, we discovered that SHD is more well-behaved than the other evaluation metrics because it considers equivalence classes when comparing structures rather than the specific DAGs. Our most surprising result was that MDL was better able to recover gold standard networks than other scoring functions given sufficient data. As expected, BDeu's performance was highly dependent on the selected *α *parameter, which can be difficult to estimate *a priori*. We also confirmed that fNML converges even with few samples. Throughout our analysis, we found AIC's behavior erratic and unpredictable. The greedy hill climbing algorithm exhibited similar behavior, so we conclude that our results hold for this algorithm, as well.

We plan to extend this work in several ways. We can use synthetic networks to more carefully control the properties of our gold standard networks. Unlike previous studies, though, we will not rely on random network generation; instead, we will handcraft a variety of networks to reflect a variety of real-world datasets. We will also incorporate other scoring metrics, such as MIT [8], and objectives, such as prediction [9], into our study.

## Competing interests

The authors declare that they have no competing interests.

## Supplementary Material

Additional file 1 **Detailed empirical results and free software packages**. The file (S1.xls) contains detailed empirical results from testing the various combinations of the scoring functions, sample sizes, and learning algorithms (sheet = results . optimal, results . greedy). It also contains a list of free software packages used in this study (sheet = Software). Click here for file
